# Biphasic Dissolution as an Exploratory Method during Early Drug Product Development

**DOI:** 10.3390/pharmaceutics12050420

**Published:** 2020-05-02

**Authors:** Daniela Amaral Silva, Jozef Al-Gousous, Neal M. Davies, Nadia Bou Chacra, Gregory K. Webster, Elke Lipka, Gordon L. Amidon, Raimar Löbenberg

**Affiliations:** 1 Faculty of Pharmacy & Pharmaceutical Sciences, University of Alberta, Edmonton, AB T6G 2E1, Canada; amaralsi@ualberta.ca (D.A.S.); ndavies@ualberta.ca (N.M.D.); 2 College of Pharmacy, University of Michigan, Ann Arbor, MI 48109, USA; jalgouso@umich.edu (J.A.-G.); glamidon@med.umich.edu (G.L.A.); 3 Faculty of Pharmaceutical Sciences, University of Sao Paulo, Sao Paulo 05508-000, Brazil; chacra@usp.br; 4 Research and Development, AbbVie Inc., North Chicago, IL 60064, USA; gregory.webster@abbvie.com; 5 Therapeutic Systems Research Laboratories, Inc., Ann Arbor, MI 48108, USA; elipka@tsrlinc.com

**Keywords:** biphasic dissolution, drug product development, buffer capacity, physiologically relevant, ibuprofen

## Abstract

Dissolution testing is a major tool used to assess a drug product’s performance and as a quality control test for solid oral dosage forms. However, compendial equipment and methods may lack discriminatory power and the ability to simulate aspects of in vivo dissolution. Using low buffer capacity media combined with an absorptive phase (biphasic dissolution) increases the physiologic relevance of in vitro testing. The purpose of this study was to use non-compendial and compendial dissolution test conditions to evaluate the in vitro performance of different formulations. The United States Pharmacopeia (USP)-recommended dissolution method greatly lacked discriminatory power, whereas low buffer capacity media discriminated between manufacturing methods. The use of an absorptive phase in the biphasic dissolution test assisted in controlling the medium pH due to the drug removal from the aqueous medium. Hence, the applied non-compendial methods were more discriminative to drug formulation differences and manufacturing methods than conventional dissolution conditions. In this study, it was demonstrated how biphasic dissolution and a low buffer capacity can be used to assess in vitro drug product performance differences. This can be a valuable approach during the early stages of drug product development for investigating in vitro drug release with improved physiological relevance.

## 1. Introduction

In the modern drug development process, a major tool used to asses a drug product’s performance is dissolution testing. The test was developed in the late 1950s/early 1960s and accepted by the United States Pharmacopeia (USP) convention in 1970 [1]. Ever since, in vitro dissolution testing has been used as a quality control (QC) test for solid oral dosage forms and it plays a critical role in enhanced product understanding [2]. The different compendial dissolution equipment includes the basket (USP apparatus 1), the paddle (USP apparatus 2), the reciprocating cylinder (USP apparatus 3), and the flow-through cell (USP apparatus 4). The latter two are used for extended-release products, whereas apparatus 2 is the most widely applied method [1]. However, compendial equipment and methods use conditions that may limit both the method’s discriminatory power and its ability to emulate aspects of in vivo dissolution. Thus, the quality control aspects of the dissolution methodologies are mostly meaningful in a commercial environment of finished drug product release. Nevertheless, during the drug product development process, in vivo predictive methods are needed for the creation of products of predictable quality [2]. In this realm, dissolution testing is a major tool used to asses a drug product’s performance.

When testing poorly soluble drugs, conditions in which the medium is not saturated should be maintained to ensure the method robustness. These are later used to test the finished product to comply with regulatory guidance [3,4]. Different strategies are often adopted to obtain such conditions throughout the dissolution test, such as the addition of solubility modifiers (e.g., surfactants) and the use of large volumes of dissolution medium among other strategies [5] that result in conditions with little physiologic resemblance. Within this context, the matter of buffer strength stands out [6].

In vivo studies demonstrate that the buffer capacity of gastrointestinal fluids is much lower than that of compendial buffers [6,7]. Not only that, but the buffer species also differ; bicarbonate is the predominant buffer species in the human small intestine [8]. This finding was linked to slower drug dissolution rates in vivo, which has important implications for the oral drug delivery of both acidic and basic drugs, and it should be considered in the in vitro dissolution studies during the drug product development process [6]. Although the intestines are buffered by bicarbonate, when possible the use of simpler buffer systems, such as phosphate, is preferred for pragmatic reasons. According to Krieg et al., the phosphate buffer concentration range needed to match ibuprofen dissolution in physiologically relevant bicarbonate buffers is 4–8 mM [9].

Furthermore, while the drug dissolves in the intestinal fluids, it is also absorbed through the gut wall. Biphasic dissolution is one of the possible approaches to assess the concurrent in vivo drug absorption process. It is composed of a two-phase system in which the simultaneous evaluation of drug dissolution and partitioning into an organic phase is studied, and it can be used as a non-compendial exploratory dissolution method. This approach was first described in the early 60s and its use has gained much attention in recent years [3,5,10,11,12,13,14,15,16,17,18].

The information obtained from the physiologically based dissolution test is used to identify what aspects of the drug substance, formulation composition, and process are most important to achieve the desired target release profile. In this way, variables that are likely to impact the drug dissolution can be identified early on in the development, allowing the ranking of formulation prototypes under physiologic-like conditions.

**Figure 1:**
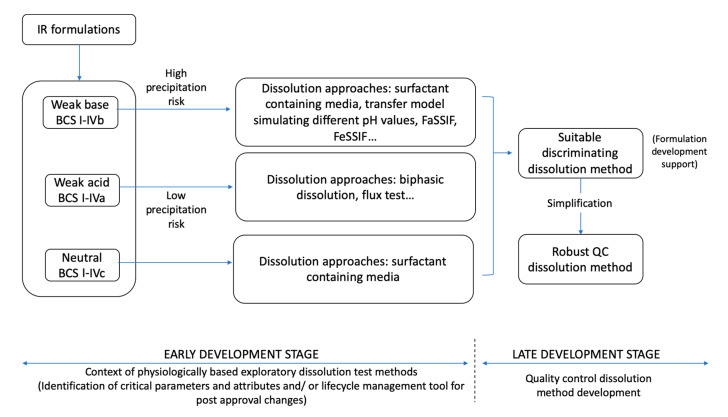
Simplified approach for dissolution method development for immediate release (IR) formulations containing acidic and basic drugs (BCS I–IV). FaSSIF: Fasted State Simulated Intestinal Fluid; FeSSIF: Fed State Simulated Intestinal Fluid; BCS: Biopharmaceutics Classification System.

As suggested by Azarmi et al., two different dissolution methods might be needed, one for formulation predictive dissolution and the other for QC purposes, which is the current practice in pharmaceutical companies [2,19]. The information obtained from early stage dissolution methods (exploratory and physiologically based) can be then used to establish appropriate discrimination of the QC method to be applied in the late development stage to critical dosage form attributes and other parameters (Figure 1).

In this exploratory study, the hypothesis was two-fold in order to evaluate both the influence of manufacturing methods and the excipient composition on the dissolution behavior of the tablets. Regarding the manufacturing process, we hypothesized that direct compression vs. wet granulation would result in different dissolution behavior, whereas excipients would create with the model drug a microclimate, also resulting in different profiles. Therefore, we screened the different tablets using compendial and physiologically based methods to identify which performance test method had the highest discriminatory power. Considering that the majority of molecules in the discovery pipeline are poorly water-soluble [20], ibuprofen (BCS IIa) was used as a model drug.

## 2. Materials and Methods

### 2.1. Materials

The ibuprofen (USP grade) was purchased from Medisca (Montreal, QC, Canada); the microcrystalline cellulose (Avicel^®^ PH-102 NF) was purchased from FMC Biopolymer (Philadelphia, PA, USA); the dicalcium phosphate dihydrate and calcium sulfate NF were purchased from PCCA Canada (London, ON, Canada). The dextrose NF was purchased from Mallinckrodt chemical (USA); the croscarmellose sodium NF was from JRS Pharma (Rosenberg, Germany); the magnesium stearate was purchased from H.L. Blachford Ltd. (Mississauga, ON, Canada); and Starch 1500 was from Colorcon (Indianapolis, IN, USA). The 1-octanol 99% pure was purchased from Acros Organics (Fair Lawn, NJ, USA). The buffer solutions were prepared with purified water (Elgastat Maxima UF and an Elgastat Option 3B water purifier by ELGA Laboratories Ltd. (Mississauga, ON, Canada)).

### 2.2. Methods

#### 2.2.1. Ibuprofen Immediate Release Formulations

The formulations used in this study differed in their excipient composition and manufacturing process (Table 1). The selection of excipient was based on their chemical characteristics in terms of basicity and acidity. Granulating BCS IIa drugs (such as ibuprofen) with acidic excipients could create a microclimate with a lower pH, reducing the drug dissolution. On the other hand, basic excipients could create a higher microclimate pH, increasing the dissolution, whereas neutral excipients would not impact the microclimate pH. Dextrose was chosen as the acidic excipient, CaSO_4_ and CaHPO_4_ as basic excipients, and microcrystalline cellulose as neutral.

In order to analyze the manufacturing method, the tablets were prepared by direct compression and wet granulation.

The direct compressed tablets (D) were prepared by mixing all the ingredients (except for the lubricant) for 6 min using a mortar and pestle until a homogenous mixture was obtained. The lubricant (magnesium stearate) was added last and mixed in for another minute to avoid the coating of the active pharmaceutical ingredient (API).

The tablets obtained by wet granulation (G) were prepared by mixing all the ingredients in the same manner as D. Ethanol 70% was used as the granulation solution and the wet powder mixture was granulated through a N60 sieve. The granules were dried for one hour in a 37 °C oven and sieved again through a N60 sieve. The lubricant (magnesium stearate) was then added into the mixture (extragranular) and blended for another minute.

All the tablets were pressed with a Carver Laboratory Press by Fred S Carver Inc. Hydraulic Equipment (Manomonee Falls, WI, USA) for 30 s at 1 metric ton.

#### 2.2.2. Dissolution Tests

All the dissolution tests were performed in triplicate using a USP apparatus II (ERWEKA, GmbH, Langen, Germany) with a 75 rpm rotation speed at 37 °C. All buffer media were filtered by vacuum and degassed in an ultrasonic bath.

##### Compendial Dissolution Method

The USP-recommended method for ibuprofen immediate release (IR) tablets is 900 mL of phosphate buffer with a pH of 7.2 (50 mM) with not less than 80% of the labeled amount dissolved in 60 min [21].

##### Non-Compendial Dissolution Methods—Physiologically Based Exploratory Methods

1. Monophasic Dissolution with a Low Buffer Capacity Medium

The literature reports that a phosphate buffer with a pH of 6.5 at concentrations between 4–8 mM matches the ibuprofen dissolution in physiologically relevant bicarbonate buffer [9]. Hence, 5 mM of phosphate buffer with a pH of 6.5 (900 mL) was used as a non-compendial and physiologically relevant dissolution medium for comparison reasons.

Samples (5 mL) were collected at specific time points (5, 10, 15, 20, 30, 45, 60 min) with media replacement after each sampling time. The amount of dissolved drug was determined by a UV-spectrophotometer at 221 nm. Since a low buffer capacity medium was being used, the pH was monitored throughout the dissolution test.

2. Biphasic Dissolution with Low Buffer Capacity Medium

Biphasic dissolution tests were performed in a 5 mM phosphate buffer with a pH of 6.5 with 100 mL of n-octanol on top. The aqueous layer mimicked the intestinal fluids and the organic layer mimicked the absorption compartment. A mini-paddle (kindly donated by Sotax AG) was mounted on the regular compendial paddle to obtain sufficient hydrodynamics in both phases.

The aqueous layer volume was also taken into consideration in order to increase the physiologic relevance. Considering the reported intestinal fluid volume of 77 mL (77 +/− 15 mL) [22], a lower volume of 200 mL was used in an attempt to better approximate that of the intestinal fluids. For comparison reasons, the dissolution experiments were also conducted at 900 mL.

Samples from the aqueous phase (5 mL) and the organic phase (1 mL) were collected at specific time points (5, 10, 15, 20, 30, 45, 60 min). The amount of the drug was determined by a UV-spectrophotometer at 221 nm for the aqueous phase and 272 nm for the organic phase. The pH of the aqueous phase was monitored throughout the dissolution test.

#### 2.2.3. Statistical Analysis

The difference between the mean dissolution values at early exposure was measured through the 90% confidence interval (CI) of difference method using the Excel Add-In DDSolver [23,24]. In order to compare the manufacturing methods, the % release at 15 min in the 5mM phosphate buffer was compared between the G and D formulations of the same composition. Furthermore, in order to compare the differences in the excipient composition, we analyzed both the early exposure (5 min) between the G formulations and at 15 min between the D formulations. The 5 min selection was based on the fact that, even though the granular disintegration/deaggregation was still happening, the microclimate effect was most meaningful and expected to be strongest at this time point. The 15 min time point was selected to be able to analyze the early exposure, but was later than disintegration time.

## 3. Results

### 3.1. Compendial Dissolution Tests

**Figure 2:**
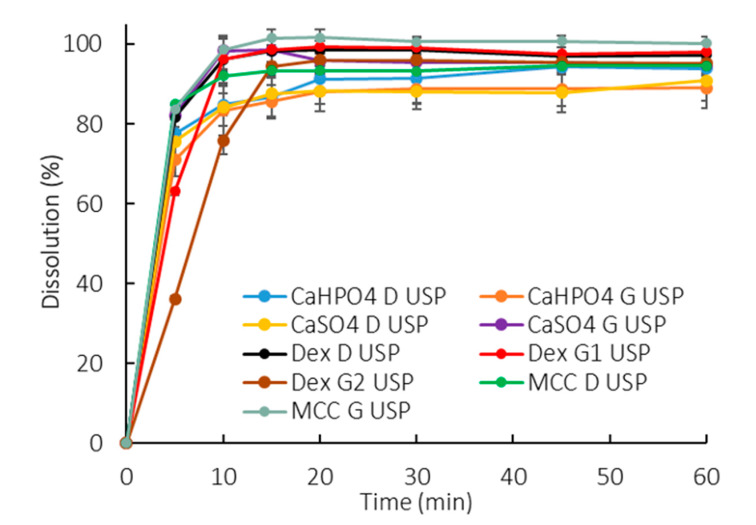
Dissolution profiles of all formulations in 900 mL of compendial buffer (50 mM phosphate buffer pH 7.2). Error bars represent the standard deviation.

All the profiles were similar in the compendial buffer, as >85% dissolved in 15 min (Figure 2). This method presented a low discriminatory power in differentiating between manufacturing methods as well as excipient compositions.

### 3.2. Non-Compendial Dissolution Tests—Physiologically Based Exploratory Methods

#### 3.2.1. Monophasic Dissolution with Low Buffer Capacity Medium

**Figure 3:**
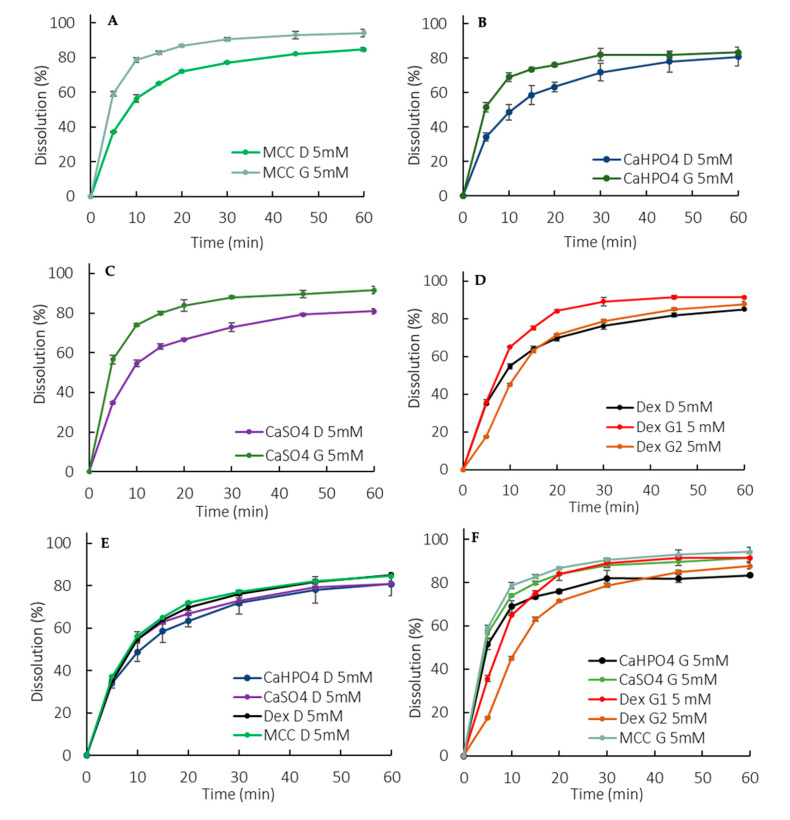
Dissolution profiles in the 5 mM phosphate buffer (900 mL). (**A**) MCC formulations, (**B**) CaHPO4 formulations, (**C**) CaSO_4_ formulations, (**D**) Dextrose formulations, (**E**) D formulations, (**F**) G formulations. Error bars represent the standard deviation.

Overall, the dissolution rate of ibuprofen was much slower in the low buffer capacity medium compared to in the USP buffer (this observation is discussed in more detail in Section 4). The release pattern among the direct compressed formulations was very similar, which points out to a dissolution controlled by the API properties rather than a formulation-driven dissolution (Figure 3E). Interestingly enough, the G formulations presented a higher rate and extent of release compared to the D formulations (Figure 3A–D), which might have been due to a reduction in the drug particle size as a consequence of the granulating process itself. The higher level of disintegrant (crosscarmellose sodium) in the granulated formulations could also have contributed to the higher release. However, it is worth noticing that a higher level of disintegrant would primarily impact the dissolution rate (especially at early time points) rather than the extent. An additional factor was that the soluble fraction of Starch1500 could have enhanced the wettability of the ibuprofen particles.

The manufacturing methods were evaluated by granulating ibuprofen, with excipients that could modulate the drug dissolution in terms of a microclimate pH (Table 1). A pronounced effect was observed at early exposure (5–10 min), particularly for the DexG2 formulation, which presented a lower release compared to the other formulations (Figure 3F).

As expected, given that ibuprofen is a weak acid dissolving in a low buffer capacity medium, a drop in pH was observed (Section 3.2.3).

#### 3.2.2. Biphasic Dissolution Test with Low Buffer Capacity Medium

##### Biphasic Dissolution with 200 mL of Aqueous Phase

With a low aqueous volume, a high interfacial area to volume ratio was obtained and, as a consequence, a rapid drug partitioning into the organic phase was observed.

**Figure 4:**
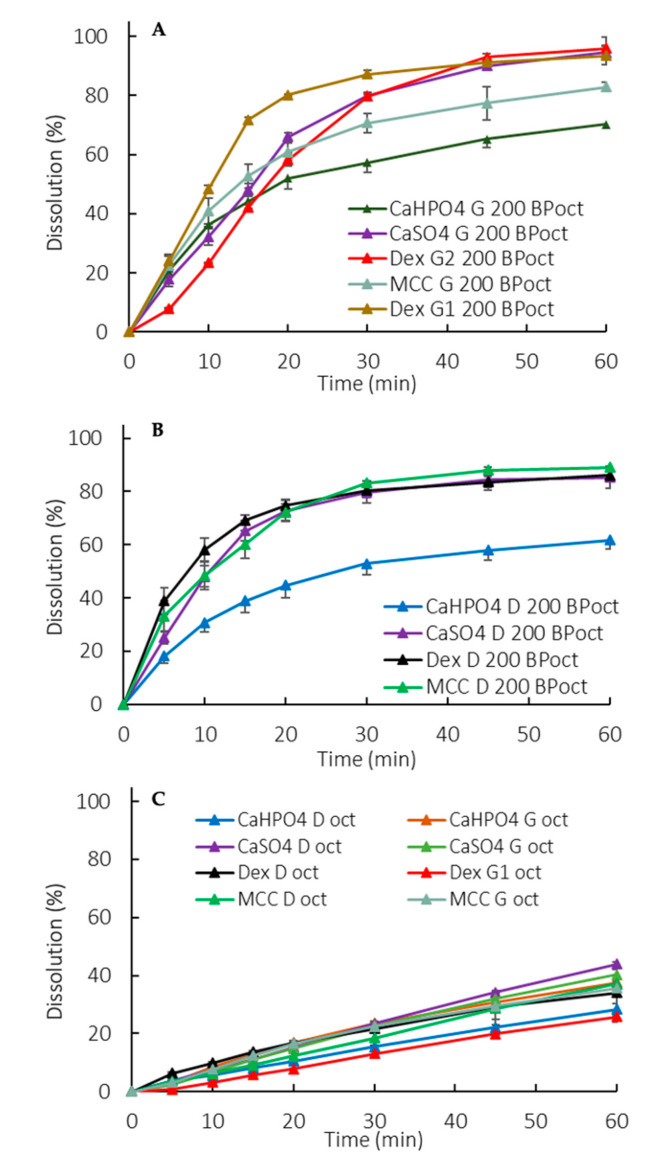
Organic phase partition profiles in a biphasic dissolution with 200 mL of aqueous media for G formulations (**A**) and D formulations (**B**) and with 900 mL of aqueous media (**C**). Oct: Octanol. Error bars represent the standard deviation.

Manufacturing method and formulation composition differences were captured in the partition profiles of the drug to the organic phase (Figure 4A,B). A lower partitioning for dextrose containing G formulations was observed at early exposure (5–15 min). An overall lower partitioning for CaHPO_4_-containing formulations (both D and G) was observed (Figure 4A,B).

##### Biphasic Dissolution with 900 mL of Aqueous Phase

**Figure 5:**
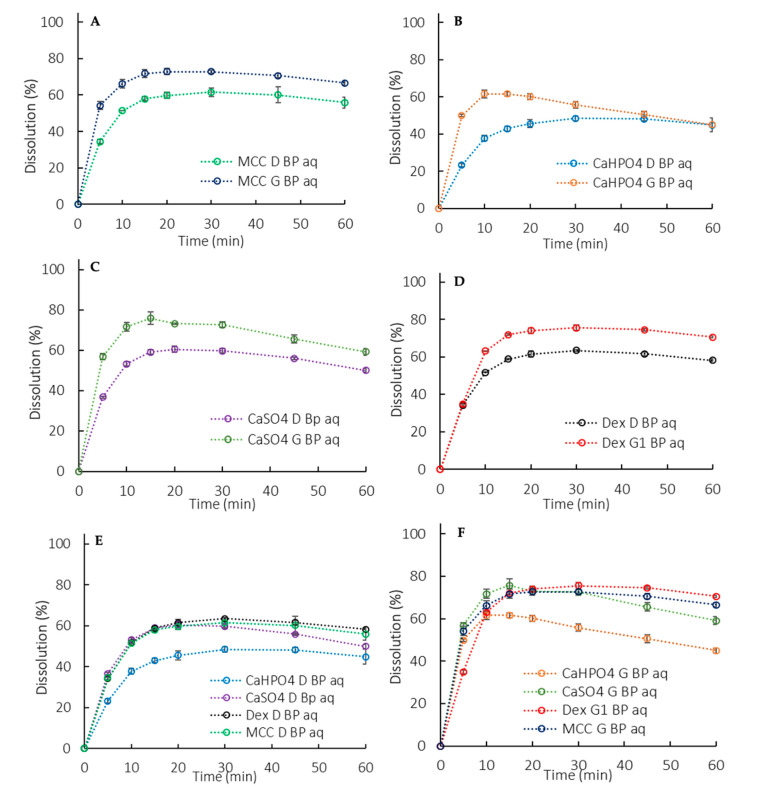
Aqueous phase dissolution profiles in a biphasic dissolution with 900 mL of aqueous media (BP aq). (**A**) MCC formulation, (**B**) CaHPO_4_ formulation, (**C**) CaSO_4_ formulation, (**D**) Dextrose formulation, (**E**) D formulations, (**F**) G formulations. Error bars represent the standard deviation.

Similarly to the monophasic dissolution (Section 3.2.1), the G formulations presented a higher rate and extent of release compared to the D formulations (Figure 5A–D). However, the higher release was not accompanied by an increased partitioning into the organic phase. Actually, the partitioning profiles of the organic phase were similar for all formulations (different excipients and manufacturing processes) (Figure 4C). Thus, in this setup, the organic phase didn’t seem suitable for formulation differentiation purposes. Instead, the organic phase added a sink to the system through the removal of the dissolved drug from the aqueous phase, reducing the pH shift observed in the monophasic dissolution (Section 3.2.3).

#### 3.2.3. Dissolution Medium pH Recovery

**Figure 6:**
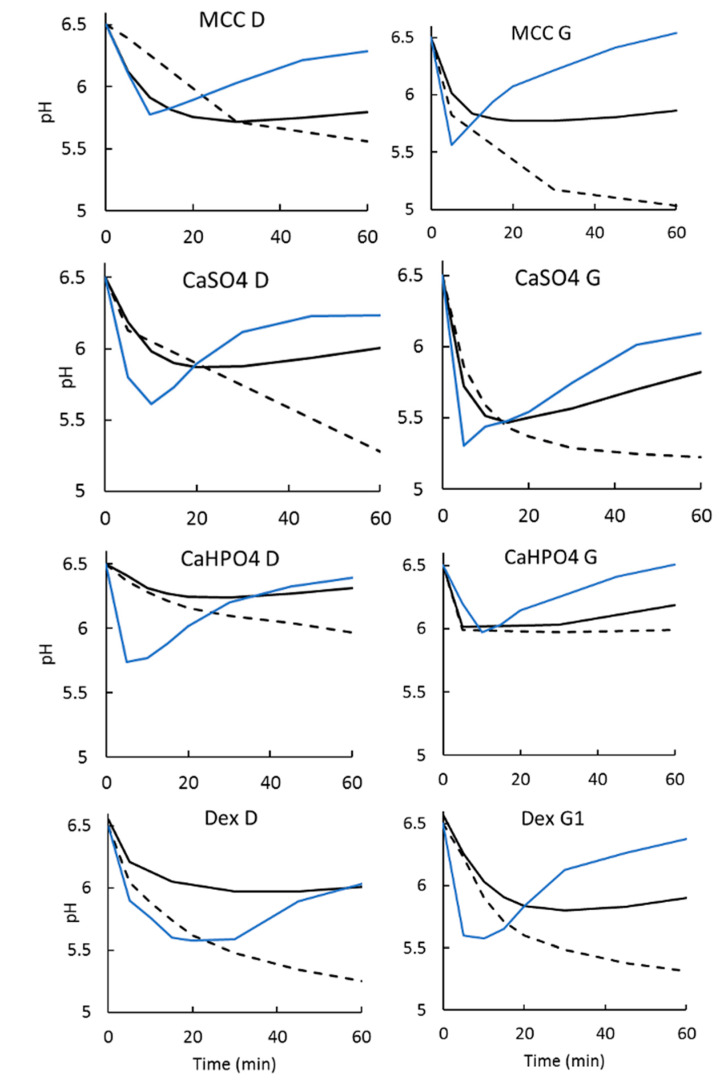
pH measurements for dissolution tests in a low buffer capacity medium. Dashed line: monophasic setup. Solid black line: biphasic setup with an aqueous layer at 900 mL. Solid blue line: biphasic setup with an aqueous layer at 200 mL.

The use of an organic layer on top of the aqueous layer assisted in the medium pH maintenance by the removal the dissolved drug from the aqueous medium. In the case of an acid, such as ibuprofen, the proton transfers to the organic phase with the drug. Hence, the pH changes that are expected when a low buffer capacity medium is used were reduced (Figure 6).

### 3.3. Statistical Analysis

The higher percentage released for the granulated tablets compared to the direct compressed ones (Figure 3A–D) was statistically relevant, as the similarity between G and D using the 90% CI was rejected for all the formulations in the 5 mM phosphate buffer.

The consistent lower release for formulations containing calcium phosphate was statistically relevant, as shown in Table 2, pointing to a possible API-excipient interaction. The suspected microclimate effect for formulations containing dextrose was also statistically significant (Table 2).

## 4. Discussion

A discriminating dissolution test is a method that can detect variations in the manufacturing process as well critical API or dosage from attributes that may have an impact on the in vivo performance of the final drug product. Physiologically based exploratory dissolution methods used in early product development often follow a generic approach [25]. Thus, in the absence of a link to in vivo drug product performance, the degree of discriminating power is often unknown. In such cases, risk assessments and prior knowledge, as well as modeling and simulation, may be helpful to guide the necessary adjustments to increase the method’s sensitivity towards certain critical API attributes, manufacturing method, and/or formulation composition [2,25].

In contrast, the pharmacopoeial experimental conditions applied in a QC setting aim at the whole amount of drug being released from the dosage form. For such purposes, a high buffer concentration (50 mM) and capacity are used. Such conditions prevent pH shifts caused by the API dissolution that could hinder or increase the dissolution, resulting in a biased data interpretation caused by the dissolution method rather than due to poor drug product performance. In the compendial conditions used in this study, a similar release was obtained for the different ibuprofen formulations, achieving the “expected release” in a QC manner (Figure 2). Nevertheless, it showed a poor discriminatory power in identifying the possible effect of critical API attributes and manufacturing methods on drug dissolution.

Accordingly, Cristofoletti and Dressman have demonstrated that in the case of ionizable compounds, the pH at the solid–liquid interface is as a key parameter in predicting the dissolution rate. The authors showed that the in vitro dissolution of ibuprofen (weak acid) in phosphate buffer is a function of the pH in the diffusion layer, which is, in turn, affected by the properties of both the drug and the medium [26]. The reported pH at the surface of the dissolving drug (ibuprofen) in a physiologically relevant bicarbonate buffer can be achieved by reducing the phosphate buffer concentration to 5 mM. Hence, using the appropriate buffer concentration for in vitro experiments would likely increase the physiological relevance of this important biopharmaceutics performance test method [26].

A rapid in vitro dissolution rate cannot be translated to the in vivo dissolution rate of ibuprofen. An in vivo study, in which the gastrointestinal (GI) drug dissolution and systemic absorption of ibuprofen was evaluated, demonstrated that the drug could still be found in the GI tract fluids even after 7 h of aspiration, pointing out that BCS II drugs may undergo a slower dissolution in the GI tract due to their low water solubility [5,6,15]. This slow dissolution rate was linked to the very low buffer capacity of luminal fluids.

This observation reflects what is going on in the drug particle diffusion layer. In highly concentrated buffer systems, the drug particle is surrounded by an abundance of the buffer’s conjugate base species. This leads to a ready neutralization in the diffusion layer around the particle, that is, the H^+^ ions formed on the dissolving drug surface are readily consumed by a buffer species. This causes the pH in the diffusion layer to be similar to the bulk solution pH, yielding a higher dissolution [9,26,27,28]. However, in a less concentrated buffer (as in the human GI tract) the neutralization is slower, which is an important physiological aspect that should be taken into account in the drug development process. Selecting the right buffer system is, therefore, of primary importance [6], since the in vitro buffer system largely affects the surface pH of the drug particle, which in turn affects its dissolution [29,30].

As previously highlighted, it has been reported that a phosphate buffer pH of 6.5 at 5 mM matches ibuprofen dissolution in physiologically relevant bicarbonate buffer [9]. The observed slower dissolution in 5 mM phosphate buffer (900 mL) compared to a USP-strength buffer (50 mM) (Figure 2 vs. Figure 3) is in line with the aforementioned in vivo findings [6]. Thus, using a low buffer capacity is an alternative approach to bring physiologically relevant components into the early stage exploratory dissolution tests. Clinically relevant specifications were not required at the time that ibuprofen was introduced to the market (late 60s/early 70s) and the developed dissolution method was a suitable test at the time.

On the other hand, as ibuprofen dissolves, the medium pH tends to decrease due to the acidic characteristics of the API (Figure 6). The pH drop observed in a low buffer capacity medium is unlikely to occur in the intestinal lumen due to neutralization mechanisms in the gut as well as the concurrent drug absorption [31]. Attempts to maintain the pH by titrating the medium with NaOH have been made [26], however it can be experimentally difficult and impractical. In cases of dissolution methods based on other buffers systems, such as bicarbonate buffer, the medium pH can be regulated by sparging the medium [8].

Combining the low buffer capacity medium with an absorptive phase adds another aspect of the in vivo gastrointestinal environment, that is, drug absorption as it dissolves in the intraluminal fluids (and in the case of an acid, such as ibuprofen, the proton transfers to the organic phase with the drug). Thus, the organic phase serves as an additional sink for the pH recovery (Figure 6).

Ibuprofen dissolution in 5 mM phosphate buffer was characterized through the D formulations, since they presented an API-controlled dissolution (Figure 3E and Figure 5E), as described by Uebbing [32]. Considering that ibuprofen is a class II drug, a reduction in particle size may increase the drug dissolution, which could be the reason for the statistically relevant higher release observed in the G formulations (Figure 3A–D and Figure 5A–D). The overall slower dissolution rate in 5 mM buffer enabled the characterization of a critical API attribute that affects in vitro dissolution.

The manufacturing method can also impact the dosage form performance. During a wet granulation process, the API and excipients come in close contact in such a way that the excipients can influence the API dissolution. After the tablet disintegrates, the drug dissolution depends also on the granules disintegration/deaggregation [33,34]. As a result, a microclimate can be created around the granulate particle, which was seen in the dextrose formulations. Since dextrose is acidic [35], the pH around the dissolving drug particle could be lower than the bulk pH, impacting the API dissolution. This effect was observed primarily at early exposure, when the granules were being deaggregated (Table 2). A lower dissolution was observed during the first time points in both the 50 mM and 5 mM phosphate buffer, and the drug partitioning into the organic phase was also affected (Figure 2, Figure 3D, Figure 4D, and Figure 5D). Early exposure is important and should be further explored in future studies. This is in accordance with Valizadeh et al., who described the impact of the microclimate on the dissolution of solid dispersions of indomethacin with different excipients, such as PEG 6000, Myrj 52, Lactose, Sorbitol, Dextrin, and Eudragit1 E100 [33].

Overall, formulations containing calcium phosphate presented a statistically relevant lower release, regardless of the manufacturing process used. In this case, another excipient-API interaction might have occurred. Even though it behaves neutrally (pH 7.4), the surface of CaHPO_4_ is alkaline [35]. Since ibuprofen is a weak acid, drug particles could have adsorbed onto the CaHPO_4_ particles due to their alkaline surface, resulting in the observed lower dissolution. It has been reported that various ions and molecules can be adsorbed onto the CaHPO_4_ surface. Furthermore, ibuprofen adsorption onto calcium phosphate beads for bone substitutes in targeted drug delivery applications has been described [36]. Incompatibilities between CaHPO_4_ and other acidic drugs, such as indomethacin and aspirin, have also been reported [35,37].

In the exploratory method used in the study, the sensitivity in discriminating dosage forms was seen in the octanol phase when applying 200 mL of the aqueous phase, whereas at 900 mL the differences were more pronounced in the aqueous phase. The low surface area to volume ratio at 900 mL and the hydrodynamics in the vessel with a paddle dissolution apparatus resulted in slower drug partitioning (drug removal) compared to at 200 mL (Figure 4A,B vs. Figure 4C) [38,39,40]. Consequently, in 900 mL partitioning is the rate-limiting step for the overall process of mass transfer between the solid, the aqueous and the octanol phases.

On the other hand, using a lower aqueous phase volume at the same rotational speed resulted in the aqueous phase experiencing a higher overall magnitude of shear stress. This might be a contributing factor to the 200 mL tending to be less discriminatory in comparing G vs. D formulations when compared on the basis of total dissolution (i.e., aqueous + organic).

Mudie et al. described the drug transport phenomenon associated with the biphasic dissolution method, assuming first-order absorption kinetics [18]. The in vitro partitioning rate coefficient (kp) represents the drug partitioning rate into the organic phase. The physiological relevance of this is that the in vitro kp approximates the in vivo absorption rate coefficient (ka), as shown in Equation (1).

(1) $$\mathrm{Kp}={\left(\frac{\mathrm{AI}}{\mathrm{Va}}\mathrm{PI}\right)}_{\mathrm{in}\;\mathrm{vitro}}=\mathrm{Ka}={\left(\frac{A}{V}\mathrm{Peff}\right)}_{\mathrm{in}\;\mathrm{vivo}}$$

PI: drug interfacial permeation rate across the aqueous and organic diffusion layers; AI: surface area of the aqueous–organic interface; Va: total volume of aqueous medium; Peff: permeation rate in vivo; A/V: area to volume ratio in vivo.

When ka and PI are known (or can be estimated), AI/Va can be adjusted so that kp and ka become similar or equal when possible. For ibuprofen, the theoretical PI reported in the literature is 23.6 × 10^−4^ cm/s [18]. Hence, the calculated kps for 900 mL (kp_900_) and 200 mL (kp_200_) are 2.1 × 10^−4^ s^−1^ and 9.35 × 10^−4^ s^−1^, respectively. In a recent human in vivo study, Hofmann and coworkers determined the real intestinal ka for ibuprofen of 2.6 × 10^−3^ s^−1^ [41]. Thus, the 900 mL underestimates the ka by a factor of 12.3, whereas the 200 mL underestimates it only by a factor of 2.8, making it much closer to the in vivo scenario. Not only that, but the pH recovery was much faster and better controlled in the 200 mL than in the 900 mL (Figure 6).

An effective drug development process aligns the best formulation strategies to obtain a suitable pharmaceutical dosage form with an adequate biopharmaceutical performance [42]. Based on this study, for an ibuprofen IR dosage form, a granulation process would be chosen over direct compression and excipients such as dextrose and CaHPO_4_ would be avoided.

The discriminatory power of biphasic dissolution is well acknowledged in the literature. Deng et al. (2017) observed a high discriminatory power in the organic phase for minor formulation changes using racecadotril as a BCS II model drug [15]. Three granule formulations of the lipophilic drug were prepared with equivalent compositions but using different manufacturing processes. The compendial tests lacked discrimination, whereas a remarkable discrimination between the granule formulations was observed in the octanol phase of the biphasic dissolution system. The test was performed in a USP II apparatus with 400 mL of phosphate buffer (50 mM, pH 6.8) as the aqueous layer and 100 mL of 1-octanol as the upper organic phase. The authors also correlated the organic phase profiles to in vivo pharmacokinetics data, which resulted in a good in vitro-in vivo correlation (IVIVC), and they concluded that “the release profiles from the organic phase could serve as an indicator for in vivo drug absorption” [15].

Several studies utilizing the biphasic system have reported its ability to obtain good IVIVCs and to be more discriminative than compendial methods [5,12,14,15,43,44,45,46,47,48]. Vangani et al. investigated the formulation changes of several compounds using the flow through apparatus (USP IV) coupled with the USP paddle apparatus in a biphasic system. An excellent rank order correlation was obtained between the in vitro release and the in vivo absorption of the drugs [43]. Al Durdunji et al. used a similar dissolution test method, i.e., USP IV coupled with USP II in a biphasic dissolution medium for a BCS II compound (Deferasirox). Similarly, the authors were able to differentiate between the formulations and establish an IVIVC [46].

Gao and coworkers reported the evaluation of several poorly soluble drugs also using a biphasic system combining USP apparatus IV (flow cell) with USP apparatus II [12,13,16,43,44,49]. Using the biphasic dissolution-partition test method, an excellent IVIVC and IVIVR (in vitro–in vivo relationship) were obtained for a number of poorly soluble drugs, such as fenofibrate, celecoxib, and ritonavir. The authors also reported little relevance of the QC dissolution test results to pharmacokinetic observations in pre-clinical and clinical studies of the prototype formulations [49]. This showcases how biphasic dissolution has the potential to reflect the in vivo environment, linking the in vitro performance to clinical relevance.

## 5. Conclusions

In light of the up-to-date mechanistic understanding of in vivo dissolution, there is a current need to rethink how product specifications and performance can be linked through physiologically relevant parameters. This study revisited the rationale of using lower buffer capacity media to increase the physiological relevance of in vitro testing. This system was demonstrated to have a superior discriminatory power regarding the manufacturing method and excipient effects. The use of an absorptive phase added a sink to the low buffer capacity media, which decreased pH shifts while the test was performed.

Hence, biphasic dissolution systems using low buffer capacity dissolution media have the potential to be used as early stage discriminatory methods to investigate the impact of excipient effects and the manufacturing method on the in vitro drug release with improved physiological relevance.

## 6. Limitations of the Study

The authors recognize that whether the difference between formulations identified by the biphasic dissolution systems with a low buffering capacity translates to in vivo difference has yet to be assessed through IVIVC. However, the biphasic dissolution tests were clearly able to discriminate between the excipient and manufacturing method, while the USP-recommended method did not discriminate between the formulations and methods.

## Figures and Tables

**Table 1:** Excipient composition of IR ibuprofen tablets prepared in-house. Formulations were named according to the diluent mixture used.

MCC D	MCC G	CaHPO_4_ D	CaHPO_4_ G	Dex D	Dex G1	Dex G2	CaSO_4_ D	CaSO_4_ G
Avicel PH102 (800 mg)	Avicel PH102 (800 mg)	Avicel PH102 (400 mg)	Avicel PH102 (400 mg)	Avicel PH102 (400 mg)	Avicel PH102 (400 mg)	Avicel PH102 (460 mg)	Avicel PH102 (400 mg)	Avicel PH102 (400 mg)
Ibuprofen (400 mg)	Ibuprofen (400 mg)	Ibuprofen (400 mg)	Ibuprofen (400 mg)	Ibuprofen (400 mg)	Ibuprofen (400 mg)	Ibuprofen (400 mg)	Ibuprofen (400 mg)	Ibuprofen (400 mg)
CS (3%)	CS (5%)	CS (3%)	CS (5%)	CS (3%)	CS (5%)	CS (5%)	CS (3%)	CS (5%)
Mg Stearate (1%)	Mg Stearate (1%)	Mg Stearate (1%)	Mg Stearate (1%)	Mg Stearate (1%)	Mg Stearate (1%)	Mg Stearate (1%)	Mg Stearate (1%)	Mg Stearate (1%)
		CaHPO_4_ (400 mg)	CaHPO_4_ (400 mg)	Dextrose (400 mg)	Dextrose (400 mg)	Dextrose (400 mg)	CaSO_4_ (400 mg)	CaSO_4_ (400 mg)
	Starch 1500 (210 mg)		Starch 1500 (210 mg)		Starch 1500 (210 mg)			Starch 1500 (210 mg)
Expected microclimate effect								
	-		↑		↓	↓↓		↑↑

MCC: microcrystalline cellulose (Avicel PH102); Dex: Dextrose; CaHPO_4_: dicalcium phosphate dihydrate; CaSO_4_: calcium sulfate; D: direct compression; G: wet granulation; CS: croscarmellose sodium; (↑ and ↑↑): increased dissolution; (↓ and ↓↓): deceased dissolution; (-): no effect.

**Table 2:** Statistical evaluation for the 90% confidence interval between different formulation compositions.

**D formulations**						
	**Dextrose**		**MCC**		**CaSO_4_**	
	**Org 200**	**Aq 900**	**Org 200**	**Aq 900**	**Org 200**	**Aq 900**
Dextrose	NA	NA	Fail	Pass	Pass	Pass
MCC	Fail	Pass	NA	NA	Fail	Pass
CaHPO_4_	Fail	Fail	Fail	Fail	Fail	Fail
**G formulations**						
	**CaHPO4**		**MCC**		**CaSO_4_**	
	**Org 200**	**Aq 900**	**Org 200**	**Aq 900**	**Org 200**	**Aq 900**
Dex G1	Pass	Fail	Pass	Fail	Pass	Fail
Dex G2	Fail	-	Fail	-	Fail	-
MCC	Pass	Pass	NA	NA	Fail	Pass
CaHPO_4_	NA	NA	Pass	Pass	Pass	Pass

Org 200: organic phase at 200 mL of aqueous phase; Aq 900: aqueous phase of biphasic test at 900 mL.
